# A rare hepatic artery variant reporting and a new classification

**DOI:** 10.3389/fsurg.2022.1003350

**Published:** 2022-08-29

**Authors:** Xiaojing Wu, Jianxiong Kang, Yuwei Liu, Guodong Sun, Ying Shi, Junqi Niu

**Affiliations:** ^1^ Department of Hepatology, First Bethune Hospital of Jilin University, Changchun, China; ^2^ General Laboratory of Human Anatomy, Changzhi Medical College, Changzhi, China

**Keywords:** accessory hepatic artery, vascular variants, phrenic artery, classification, case report

## Abstract

Variations of the hepatic artery are very common, but they greatly increase the difficulty of surgery and the risk of complications in perihepatic surgeries such as liver transplantation, liver segmentectomy, and gastroduodenal surgery. Thus, it is important to precisely define the type of hepatic artery variant before surgery. However, there are often rare variants that cannot be defined with existing classifications. For example, the type of hepatic artery variant in the current case could not be classified with conventional classifications, and no such variation has been reported to date, involving two accessory left hepatic arteries from the common hepatic and left inferior phrenic arteries, respectively. Based on the existing 3DCT technology and the CRL classification method, which is applicable to the most common hepatic artery variants, we reviewed many rare variant types and proposed a new classification method (ex-CRL classification) for hepatic artery variations that do not fit the classic scope. The ex-CRL classification can accurately classify the vast majority of rare cases in the literature, greatly compensates for the limitations of current hepatic artery classifications, improves the generalization and understanding of rare cases, and reduces surgical complications.

## Introduction

The common hepatic artery (CHA) is one of the main arterial supplies of the liver, gallbladder, lesser omentum, and gastroduodenal region, and its branches and variations are complicated. Interestingly, normal patterns occur only in 42%–75.7% of cases (1, 2). Aberrant arteries may be accidentally injured during surgical procedures resulting in severe haemorrhage and other fatal complications (3–11). A thorough knowledge of possible variations in branching, courses, and distribution of the vessels supplying the liver and gallbladder is essential. Therefore, many variations of the hepatic artery have been described and classified, among which the classic classifications are Michel's 10 types and Hiatt's 6 types (1, 8). However, despite the many classifications, more than 10% of hepatic arteries still cannot be classified. In recent years, with the wide application of 3D visualization technology (3DVT) (9–11), Yan et al. (12) presented the CRL classification, which succinctly describes the hepatic artery and its branches and covers a wider range of arterial variants. However, some rare variants still cannot be categorized by the CRL classification, and these variations are more likely to be unidentified and injured. These rare types have been reported in the form of individual cases for many years, lacking systematic classification and generalization. The aim of this study was to report a variation of the hepatic artery not in any of the hepatic classifications. Thereafter, based on CRL classification the study puts forward an extended version (ex-CRL classification) specifically for some rare cases.

## Case presentation

The variation of the hepatic artery was found during a routine dissection of an adult male cadaver for teaching purposes at Changzhi Medical College in 2020. In compliance with the confidentiality of the donor, we have not obtained the medical history and related information of the cadaver. The specimens were perfused with 5% formaldehyde and 10% alcohol, and fixed with 4% formaldehyde solution. The structure of the specimen was clear and intact.

**Figure 1:**
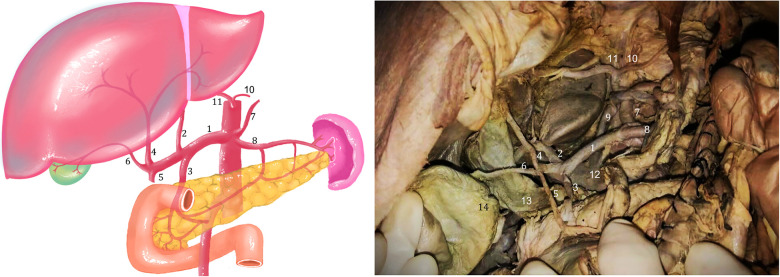
Photograph and schematic diagram of hepatic artery variation. 1. common hepatic artery (CHA); 2. accessory left hepatic artery (aLHA); 3. gastroduodenal artery (GDA); 4. proper hepatic artery (PHA); 5. right gastric artery 6. cystic artery; 7. left gastric artery; 8. splenic artery; 9. left gastric vein; 10. phrenic artery; 11. another accessory left hepatic artery (aLHA); 12. portal vein; 13. common hepatic duct (CHD); 14. Gallbladder.

CHA, originated from the celiac trunk (CT), which travelled behind the stomach and gave off three branches as follows: the accessory left hepatic artery (aLHA), proper hepatic artery (PHA) and gastroduodenal artery (GDA). The aLHA arose 22.0 mm distal to the origin of the CHA, travelling behind the PHA and finally entering into segment IV, posterior to the common hepatic duct (CHD), and its one bunch ran through the umbilical fissure. The PHA then travelled to the right about 11.4 mm and divided into the right gastric artery and cystic artery, and then ran superiorly about 34.2 mm and entered the liver through the porta hepatis (Figure 1).

In addition, the abdominal aorta gave off the left inferior phrenic artery at about 23.0 mm above the CT and was associated with agenesis of the right inferior phrenic artery. The left inferior phrenic artery provided the second left accessory hepatic artery. The second left accessory hepatic artery partially joined the left interlobar artery of the left lobe through the venous ligament and partially supplied the left lobe of the liver, forming a small branch supplying the falcine ligament and partially supplying the right diaphragm.

## Discussion

To the best of our knowledge, there has been no case report of the hepatic artery arising from the inferior phrenic artery and replacing the right inferior phrenic artery. Only Covey et al. (13) reported the origin of the hepatic artery from the phrenic artery. They collected arterial anatomical data from 600 cases of visceral angiography from May 1996 to October 2000, and found that one case of rRHA and aRHA each originated from the right phrenic artery. Thereafter, Liang (14) searched the previous literature and analysed 21 studies, including that of Covey et al., with a total of 10,966 cases, and only 2 cases reported by Covey et al. noted that the hepatic artery originated from the phrenic artery. Jin (15) reviewed 10, 211 cases in 21 articles and found that only 2 cases originating from the phrenic artery were reported by Covey et al. Therefore, the origin of the accessory hepatic artery from the phrenic artery reported in this case is extremely rare and noteworthy.

In addition, another accessory hepatic artery in the current case originated from the common hepatic artery. Futara (16) dissected 110 cases and found that in 5 cases (4.5%), the left accessory hepatic artery originated from the common hepatic artery, while Pai (17) collected autopsy data from 72 cadavers between 2000 and 2005 and found only one (1.38%) variant of the accessory hepatic artery originating from CHA, which was much lower than the expected incidence (4.5%).

However, we think about reasons why this variant has not been previously described, except that it has an extremely low incidence and may also be associated with limited 2D CT techniques. 2D technologies very test the spatial imagination of surgeons (18), except for conventional variants such as left gastric artery and superior mesenteric artery, other rare variants are difficult to find. In this case, the accessory left hepatic artery originating from the inferior phrenic artery is short and concealed, and we only exposed its course completely after excising part of the left lobe, so it is easily overlooked.

In the current case, the hepatic artery that replaced the right inferior phrenic artery was thicker than the left inferior phrenic artery, and has a wider range of blood supply. This variation is likely to cause ischaemia and dysfunction of the right diaphragm during left hepatic lobectomy, lymph node dissection around a gastric cancer, infusion of chemotherapeutic drugs for hepatocellular carcinoma, and vascular embolisation. In addition, the accessory hepatic artery may not be fully visualised during selective hepatic arteriography, and such vascular variants interfere with effective tumour control by transcatheter arterial chemoembolisation (19). If a brain-dead donor of this variant type is encountered during liver transplantation, all blood vessels entering the liver should be preserved as long as possible. During vascular reconstruction, it can be flexibly determined according to the recipient's vascular condition, and blood vessels with an appropriate diameter and in good condition are selected for end-to-end anastomosis with the donor. When the recipient has poor vascular conditions such as the common hepatic artery and gastroduodenal artery due to the underlying liver disease, but the splenic artery is in good condition, an anastomosis with the easily separated splenic artery is selected; if the donor liver cannot obtain arterial blood supply *in situ*, The donor iliac vessels were selected to bypass the hepatic artery with the abdominal aorta (20). If this type of variant is a living donor, due to the presence of the double accessory left hepatic arteries, a right half liver can also be considered for living donor liver transplantation. Of course, it is still controversial as to whether multiple arteries need routine reconstruction, so full surgical protocol should also be made with adequate preoperative evaluation (21).

Although the variants of the two combinations in this case are extremely rare, these variants are complex and easy to ignore and could lead serious intraoperative complications. Therefore, the accumulation of knowledge regarding this type of variant has important anatomical significance for surgery and interventional therapy.

**Figure 2:**
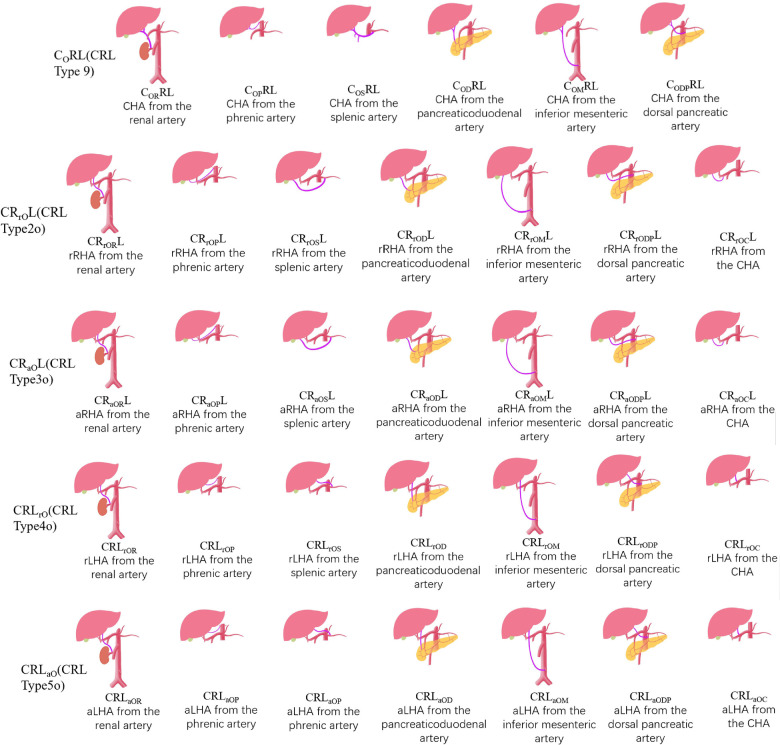
Types and diagrams in ex-CRL classification system.

In recent years, with the development of 3DCT technology, 3DVT is clearer and more accurate in imaging blood vessels compared to 2D imaging (22). 3DVT is widely used in preoperative evaluation and provides a clearer understanding of the origin and course of CHA, LHA, and RHA. Therefore, there is also a need for a new type of classification that is more compatible with 3DCT to describe and classify hepatic arteries more concisely. The CRL classification is a new classification developed based on 3DCT technology, with each hepatic artery described in detail. It conforms to the current requirements for preoperative evaluation and is suitable for most routine variants. However, it is currently not specific for rare cases. Therefore, we propose a new classification, the ex-CRL classification (Figure 2), on the basis of the CRL classification to name and classify variants that cannot be classified by the CRL classification.

### CRL classification rules

CRL describes the origin of CHA, RHA, and LHA, with CRL being the abbreviation of their initials. CRL describes normal origins; that is, CHA originates from CT, while RHA and LHA originate from PHA. Thereafter, the origins of other arteries are marked with capital letters corresponding to their arteries. For example, C_S_RL means that the origin of CHA has changed, not from CT but from SMA, while the origins of RHA and LHA remain changed. The replaced or accessory hepatic arteries need to be represented by lowercase letters “r” or “a”. For example, CR_rG_L_aA_ means that CHA originates from CT, rRHA originates from GDA, and aLHA originates from the aorta. The CRL classification is detailed in Table 1.

**Table 1:** Types in CRL classification system.

Classifification	Description
Type 1 (CRL)	CHA from coeliac trunk; LHA and RHA from PHA
Type 2 (CR_r—_L)	rRHA only
2a (CR_rA_L)	rRHA from aorta
2c (CR_rC_L)	rRHA from coeliac trunk
2g (CR_rG_L)	rRHA from GDA
2s (CR_rS_L)	rRHA from SMA
2o (CR_rO_L)	rRHA from other arteries
Type 3 (CR_a—_L)	aRHA
3a (CR_aA_L)	aRHA from aorta
3c (CR_aC_L)	aRHA from coeliac trunk
3g (CR_aG_L)	aRHA from GDA
3s (CR_aS_L)	aRHA from SMA
3o (CR_aO_L)	aRHA from other arteries
Type 4 (CRL_r—_)	rLHA
4g (CRL_rG_)	rLHA from GDA
4l (CRL_rL_)	rLHA from LGA
4o (CRL_rO_)	rLHA from other arteries
Type 5 (CRL_a—_)	aLHA
5g (CRL_aG_)	aLHA from GDA
5l (CRL_aL_)	aLHA from LGA
5o (CRL_aO_)	aLHA from other arteries
Type 6 (CR_r_L_r_)	rRHA and rLHA (type 2 + type 4)
CR_r__L_r__	rRHA from aorta, coeliac trunk, GDA, SMA or other arteries; rLHA from GDA, LGA or other arteries
Type 7 (CR_r_L_a_ or CR_a_L_r_)	rRHA and aLHA (type 2 + type 5), or aRHA and rLHA (type 3 + type 4)
7a (CR_r__L_a__)	rRHA from aorta, coeliac trunk, GDA, SMA or other arteries; aLHA from GDA, LGA or other arteries
7r (CR_a__L_r__)	aRHA from aorta, coeliac trunk, GDA, SMA or other arteries; rLHA from GDA, LGA or other arteries
Type 8 (CR_a_L_a_)	aRHA and aLHA (type 3 + type 5)
CR_a__L_a__	aRHA from aorta, coeliac trunk, GDA, SMA or other arteries; aLHA from GDA, LGA or other arteries
Type 9 (C_RL)	rCHA not from coeliac trunk
9a (C_A_RL)	CHA from aorta
9l (C_L_RL)	CHA from LGA
9s (C_S_RL)	CHA from SMA
9o (C_O_RL)	CHA from other arteries

### ex-CRL classification rules

The ex-CRL classification is suitable for a small number of variants outside the CRL classification. Other arteries in the CRL classification are uniformly represented by “O”. For example, CR_aO_L, indicates that aRHA originates from other arteries other than AA, CT, PHA, LGA, GDA, or SMA, while ex-CRL subdivides other arteries that have appeared in the past, such as “OR”, which indicates origin from the renal artery; “ OP”, from the phrenic artery; “OS”, from the splenic artery; “OC”, from CHA; “OD”, from the pancreaticoduodenal artery; “OM”, from the inferior mesenteric artery; and “ODP” from the dorsal pancreatic artery. Detailed rules for the ex-CRL classification are shown in Tables 2, 3.

**Table 2:** Terminology and Nomenclature of CRL and ex-CRL classification.

Label	Description
C	origin of CT
R	origin of RHA
L	origin of LHA
O	other arteries
OR	originating from the renal artery
OP	originating from the phrenic artery
OS	originating from the splenic artery
OC	originating from the CHA
OD	originating from the pancreaticoduodenal artery
OM	originating from the inferior mesenteric artery
ODP	originating from the dorsal pancreatic artery

**Table 3:** ex-CRL classification.

Classifification	Description
C_O_RL (CRL Type 9)	CHA from other arteries
C_OR_RL	CHA from the renal artery
C_OP_RL	CHA from the phrenic artery
C_OS_RL	CHA from the splenic artery
C_OD_RL	CHA from the pancreaticoduodenal artery
C_OM_RL	CHA from the inferior mesenteric artery
C_ODP_RL	CHA from the dorsal pancreatic artery
CR_rO_L (CRL Type2o)	rRHA from other arteries
CR_rOR_L	rRHA from the renal artery
CR_rOP_L	rRHA from the phrenic artery
CR_rOS_L	rRHA from the splenic artery
CR_rOC_L	rRHA from the CHA
CR_rOD_L	rRHA from the pancreaticoduodenal artery
CR_rOM_L	rRHA from the inferior mesenteric artery
CR_rODP_L	rRHA from the dorsal pancreatic artery
CR_aO_L (CRL Type3o)	aRHA from other arteries
CR_aOR_L	aRHA from the renal artery
CR_aOP_L	aRHA from the phrenic artery
CR_aOS_L	aRHA from the splenic artery
CR_aOC_L	aRHA from the CHA
CR_aOD_L	aRHA from the pancreaticoduodenal artery
CR_aOM_L	aRHA from the inferior mesenteric artery
CR_aODP_L	aRHA from the dorsal pancreatic artery
CRL_rO_ (CRL Type4o)	rLHA from other arteries
CRL_rOR_	rLHA from the renal artery
CRL_rOP_	rLHA from the phrenic artery
CRL_rOS_	rLHA from the splenic artery
CRL_rOC_	rLHA from the CHA
CRL_rOD_	rLHA from the pancreaticoduodenal artery
CRL_rOM_	rLHA from the inferior mesenteric artery
CRL_rODP_	rLHA from the dorsal pancreatic artery
CRL_aO_ (CRL Type5o)	aLHA from other arteries
CRL_aOP_	aLHA from the renal artery
CRL_aOP_	aLHA from the phrenic artery
CRL_aOS_	aLHA from the splenic artery
CRL_aOC_	aLHA from the CHA
CRL_aOD_	aLHA from the pancreaticoduodenal artery
CRL_aOM_	aLHA from the inferior mesenteric artery
CRL_aODP_	aLHA from the dorsal pancreatic artery
Combinatorial variation	Two or more accessory artery, replaced artery or middle hepatic artery
CR_r1_,r2__L_	two rRHAs; eg: "CR_r1A,r2L_L", means"rRHA1 form aorta, rRHA2 from LGA"
CR_a1_,a2__L_	two aRHAs; eg: "CR_a1A,a2L_L", means"aRHA1 form aorta, aRHA2 from LGA"
CR_L_r1_,r2__	two rLHAs; eg: "CRL_r1A,r2L_", means"rLHA1 form aorta, rLHA2 from LGA"
CR_L_a1_,a2__	two aLHAs; eg: "CRL_a1A,a2L_", means"aLHA1 form aorta, aLHA2 from LGA"
C_R_L_	CHA, aRHA or rRHA, aLHA or rLHA from three different arteries, and CHA not from coeliac trunk
C_R_M_L_	MHA is present and not from PHA

In addition, ex-CRL complements multiple replaced or accessory arteries, as well as in the case of MHA. It uses “a1”, “a2”, “r1”, and “r2” to represent multiple replaced or accessory hepatic arteries. As reported in this case, aLHA1 originates from CHA, and aLHA2 originates from the phrenic artery, and is represented by CRL_a1OC, a2OP_, which succinctly and clearly illustrates the variation of the two aLHAs. In addition, MHA is not uncommon in variants. When MHA originates from PHA, RHA, or LHA, it does not need special notation, because it is close to the hepatic portal and bifurcates in advance before entering the liver to supply the fourth segment of liver. However, when MHA originates from other arteries and is difficult to be classified as RHA or LHA, special labeling is required. For example, Gündoğdu et al. (23) described MHA originating from the pancreaticoduodenal artery, and aRHA originating from the dorsal pancreatic artery in 2021, which can be represented by CR_aODP_M_OP_L.

Finally, cases with variations in CHA, RHA, and LHA have been supplemented. For example, CHA from AA, aLHA from LGA, aRHA from SMA, were reported by Gruttadauria et al. (24) in 2001. However, the current case cannot be classified with the CRL classification, but can be expressed as C_A_R_aS_L_aL_ based on the ex-CRL classification.

### Evaluation of the ex-CRL classification

Different classifications were used to describe rare cases in the previous literature (Table 4). We reviewed the literature on hepatic artery variants from 1994 to 2021, and finally retained 16 studies (2878 cases) describing rare variants, including 17 rare hepatic arterial variants that cannot be described by common classification methods. We found that the classic Hiatt and Michel classifications were unsuitable for these rare and complex classifications, and their coverage by the CRL classification was relatively improved, but the specificity for these rare cases was not high. For example, rRHA described by Abdullah et al. (33) in 2006 originated from the inferior mesenteric artery, which is classified as CR_rOM_L by ex-CRL, and rRHA described by Imam et al. (25) in 2021 was derived from the pancreaticoduodenal artery, which was classified as CR_rOD_L by ex-CRL, but both cases are represented by CRL classification as “2o (CR_rO_L)”. The CRL classification does not clearly describe this part of “other arteries”, making it necessary to use ex-CRL for reclassification.

**Table 4:** Evaluation of Ex-CRL classification.

Author	Year	Total cases	Case	Michels	Hiatt	CRL classifification	ex-CRL
Current study	2022	1	aLHA_1_ from CHA, aLHA_2_ from phrenic artery	n.d.	n.d.	n.d.	**CRL_a1OC,a2OP_**
Imam et al (25)	2021	241	aRHA from pancreaticoduodenal artery	n.d.	n.d.	3o (CR_aO_L)	**CR_aOD_L**
			rRHA from pancreaticoduodenal artery	n.d.	n.d.	2o (CR_rO_L)	**CR_rOD_L**
			aRHA from pancreaticoduodenal artery, rLHA from LGA	n.d.	n.d.	7r (CR_aL_r)	**CR_aOD_L_rL_**
			rRHA from pancreaticoduodenal artery, rLHA from LGA	n.d.	n.d.	6(CR_r_L_r_)	**CR_rOD_L_rL_**
Gündoğdu et al (23)	2021	1	aRHA from dorsal pancreatic artery, MHA from pancreaticoduodenal artery	n.d.	n.d.	n.d.	CR_aODP_M_OP_L
De Blasi et al (26)	2019	1	aRHA from splenic artery, aLHA from LGA	n.d.	n.d.	8 (CR_a_L_a_)	**CR_aL_L_aOS_**
Darsan et al (27)	2019	2	aRHA from renal artery	n.d.	n.d.	3o (CR_aO_L)	**CR_aOR_L**
Al Zahrani et al (28)	2017	1	aRHA form splenic artery	n.d.	n.d.	3o (CR_aO_L)	**CR_aOS_L**
Caruso et al (29)	2016	1	aRHA from splenic artery, aLHA from LGA	n.d.	n.d.	8 (CR_a_L_a_)	**CR_aL_L_aOS_**
Dandekar et al (30)	2015	60	aRHA from CHA	n.d.	n.d.	3o (CR_aO_L)	**CR_aOC_L**
Polguj et al (31)	2010	1	aRHA from CHA	n.d.	n.d.	3o (CR_aO_L)	**CR_aOC_L**
Wang et al (32)	2007	1	CHA from renal artery	n.d.	n.d.	9o (C_O_RL)	**C_OR_RL**
Abdullah et al (33)	2006	932	rRHA from inferior mesenteric artery	n.d.	n.d.	2o (CR_rO_L)	**CR_rOM_L**
Kishi et al (34)	2004	223	aRHA from superior pancreaticoduodenal artery	n.d.	n.d.	3o (CR_aO_L)	**CR_aOD_L**
Covey et al (13)	2002	600	rRHA from right phrenic artery	n.d.	n.d.	2o (CR_rO_L)	**CR_rOP_L**
			aRHA from right phrenic artery	n.d.	n.d.	3o (CR_aO_L)	**CR_aOP_L**
Gruttadauria et al (24)	2001	701	aRHA from renal artery	n.d.	n.d.	3o (CR_aO_L)	**CR_aOR_L**
			rRHA from CHA	n.d.	n.d.	2o (CR_rO_L)	**CR_rOC_L**
			CHA from AA, aLHA from LGA, aRHA from SMA	n.d.	n.d.	n.d.	**C_A_R_aS_L_aL_**
Futara et al (16)	2001	110	aLHA from CHA	n.d.	n.d.	5o (CRL_aO_)	**CRL_aOC_**
Hardy et al (8)	1994	2	rLHA from right phrenic artery	n.d.	n.d.	4o (CRL_rO_)	**CRL_rOP_**
Braun et al (35)	1991	1	rRHA from right renal artery	n.d.	n.d.	2o (CR_rO_L)	**CR_rOR_L**

The CRL classification can classify some rare cases, but it lacks specificity and cannot describe them accurately. When the case base is large, the number of rare cases will increase; thus, attention is required for these rare cases. The application of ex-CRL, as an extension of the CRL classification, is conducive to improving the understanding and classification of rare variants and reducing the occurrence of surgical complications.

However, all classification methods, including ex-CRL classification, describe the origin of the variant artery, but lack the description of the path of the variant artery. Even if they originate from the same artery, their bifurcation planes and paths may be different. For example, two cases of aRHA originating from the renal artery reported by Darsan (27) in 2019 originated from the proximal end of the renal artery near the aorta and the distal end of the renal artery near the renal hilum, and their ascending paths were also different. This requires careful observation while performing an analysis of the detailed path of the artery to ensure that rapid and precise judgements are made in a limited surgical field of view.

Variations of the hepatic artery and surrounding blood vessels are associated with increased surgical difficulty and complications (36). For example, the accessory hepatic artery varies in liver transplant donors. If the accessory hepatic artery is ligated, it will lead to local arterial ischaemia, which may compromise liver function. If the accessory hepatic artery is preserved, it will lead to an increase in the number of arterial reconstructions and anastomosis, and a longer warm ischaemia time, which has been proven to be a risk factor for hepatic artery thrombosis (37). However, if the recipient has a complex hepatic artery vascular variation, due to the small and complex blood vessels of the hepatic artery, it will increase the incidence of hepatic artery thrombosis, hepatic artery stenosis, hepatic artery haemorrhage, or other nonvascular complications, including bile duct stenosis after liver transplantation as well as liver abscesses (38). Additionally, in pancreaticoduodenectomy, perihepatic arterial variation increases the risk of compromised hepatic arterial supply, which may lead to unintended bleeding or ischaemia, as well as an increased risk of biliary anastomotic leakage, transient liver function disorders, and, in some patients, liver failure (39).

In addition to invasive angiographic techniques, and noninvasive vascular imaging techniques, 3D reconstruction techniques such as volume rendering technique (VRT) and curved planar reformation (CPR) have rapidly evolved, they allow stereoscopic imaging of splanchnic arteriovenous and other conduits, reflecting the complete vessel diameter, length, course, and positional relationships of surrounding soft tissues and organs (40, 41). We expect that 3D visualization technology and CRL classification will become more widespread and routinely used for preoperative evaluation and surgical simulation of perihepatic surgery. Improved understanding of vascular anatomy, imaging, and classification of hepatic arteries will facilitate the development of individualized surgical protocol and avoid complications, while also expecting that more rare types of new hepatic artery variants will be discovered.

## Conclusion

Comprehensive knowledge, accumulation, and classification of hepatic arterial variant types can greatly reduce surgical complications, and the ex-CRL classification can be used for hepatic arterial variants that cannot be classified with conventional methods.

## Data Availability

The original contributions presented in the study are included in the article/Supplementary Material, further inquiries can be directed to the corresponding author/s.

## References

[1] Michels NA. Newer anatomy of the liver and its variant blood supply and collateral circulation. Am J Surg. (1966) 112(3):337–47. 10.1016/0002-9610(66)90201-7 5917302

[2] Németh K Deshpande R Máthé Z Szuák A Kiss M Korom C Extrahepatic arteries of the human liver - anatomical variants and surgical relevancies. Transpl Int. (2015) 28(10):1216–26. 10.1111/tri.12630 26152659

[3] Takagi K Domagala P Polak WG Ijzermans JNM Boehnert MU. Right posterior segment graft for living donor liver transplantation: a systematic review. Transplant Rev (Orlando). (2020) 34(1):100510. 10.1016/j.trre.2019.100510 31495539

[4] Takeda M Sakamoto S Uchida H Yoshimura S Shimizu S Hirata Y Technical considerations in liver transplantation for biliary atresia with situs inversus. Liver Transpl. (2019) 25(9):1333–41. 10.1002/lt.25484. 31063622

[5] Albers BK Khanna G. Vascular anomalies of the pediatric liver. Radiographics. (2019) 39(3):842–56. 10.1148/rg.2019180146 31059404

[6] Baker TB Zimmerman MA Goodrich NP Samstein B Pomfret EA Pomposelli JJ Biliary reconstructive techniques and associated anatomic variants in adult living donor liver transplantations: the adult-to-adult living donor liver transplantation cohort study experience. Liver Transpl. (2017) 23(12):1519–30. 10.1002/lt.24872 28926171 PMC5818204

[7] Schroering JR Kubal CA Fridell JA Hathaway TJ Robinson RC Mangus RS. Impact of variant donor hepatic arterial anatomy on clinical graft outcomes in liver transplantation. Liver Transpl. (2018) 24(10):1481–4. 10.1002/lt.25316 30054968 PMC6298596

[8] Hardy K Jones R. Hepatic artery anatomy in relation to reconstruction in liver transplantation: some unusual variations. Aust N Z J Surg. (1994) 64(6):437–40. 10.1111/j.1445-2197.1994.tb02248.x 8010909

[9] Bogomolova K Hierck BP Looijen AEM Pilon JNM Putter H Wainman B Stereoscopic three-dimensional visualisation technology in anatomy learning: a meta-analysis. Med Educ. (2021) 55(3):317–27. 10.1111/medu.14352 32790885 PMC7984401

[10] Nation H Kaliski D Ortiz A. Narrated dissection videos and peer-mentoring to enhance anatomy performance of underrepresented minority students in physical therapy education. Anat Sci Educ. (2020) 13(6):794–9. 10.1002/ase.1971 32384222

[11] Wainman B Wolak L Pukas G Zheng E Norman GR. The superiority of three-dimensional physical models to two-dimensional computer presentations in anatomy learning. Med Educ. (2018) 52(11):1138–46. 10.1111/medu.13683 30345680

[12] Yan J Feng H Wang H Yuan F Yang C Liang X Hepatic artery classification based on three-dimensional ct. Br J Surg. (2020) 107(7):906–16. 10.1002/bjs.11458 32057096

[13] Covey A Brody L Maluccio M Getrajdman G Brown K. Variant hepatic arterial anatomy revisited: digital subtraction angiography performed in 600 patients. Radiology. (2002) 224(2):542–7. 10.1148/radiol.2242011283 12147854

[14] Liang Y Li E Min J Gong C Wu L. Rare anatomic variation of the right hepatic artery and accessory right hepatic artery supplying hepatocellular carcinoma: a case report and literature review. Medicine (Baltimore). (2017) 96(39):e8144. 10.1097/md.0000000000008144 28953651 PMC5626294

[15] Jin W Dong M Pan J Zhang Q Li M Guo D Rare combined variations of accessory left hepatic artery and accessory right hepatic artery: a case report and literature review. Surg Radiol Anat. (2020) 42(4):443–7. 10.1007/s00276-019-02396-4 31811353

[16] Futara G Ali A Kinfu Y. Variations of the hepatic and cystic arteries among Ethiopians. Ethiop Med J. (2001) 39(2):133–42. 11501290

[17] Pai R Hunnargi A Srinivasan M. Accessory left hepatic artery arising from common hepatic artery. Indian J Surg. (2008) 70(2):80–2. 10.1007/s12262-008-0021-0 23133027 PMC3452402

[18] Bijlstra OD Broersen A Oosterveer TTM Faber RA Achterberg FB Hurks R Integration of three-dimensional liver models in a multimodal image-guided robotic liver surgery cockpit. Life (Basel). (2022) 12(5):667. 10.3390/life12050667 PMC9144252 35629335

[19] Kim H-C Chung JW Lee W Jae HJ Park JH. Recognizing extrahepatic collateral vessels that supply hepatocellular carcinoma to avoid complications of transcatheter arterial chemoembolization. Radiographics. (2005) 25(Suppl 1):S25–39. 10.1148/rg.25si055508 16227494

[20] Li P Thorat A Jeng L Yang H Li M Yeh C Hepatic artery reconstruction in living donor liver transplantation using surgical loupes: achieving low rate of hepatic arterial thrombosis in 741 consecutive recipients-tips and tricks to overcome the poor hepatic arterial flow. Liver Transpl. (2017) 23(7):887–98. 10.1002/lt.24775 28422392

[21] Thenappan A Jha RC Fishbein T Matsumoto C Melancon JK Girlanda R Liver allograft outcomes after laparoscopic-assisted and minimal access live donor hepatectomy for transplantation. Am J Surg. (2011) 201(4):450–5. 10.1016/j.amjsurg.2010.10.007 21421098

[22] Fang C Tao H Yang J Fang Z Cai W Liu J Impact of three-dimensional reconstruction technique in the operation planning of centrally located hepatocellular carcinoma. J Am Coll Surg. (2015) 220(1):28–37. 10.1016/j.jamcollsurg.2014.09.023 25456781

[23] Gündoğdu E Kebapçı M. Two novel hepatic arterial variations in a living liver donor detected by multidetector computed tomography angiography. Surg Radiol Anat. (2021) 43(8):1385–9. 10.1007/s00276-021-02730-9 33682016

[24] Gruttadauria S Foglieni C Doria C Luca A Lauro A Marino I. The hepatic artery in liver transplantation and surgery: vascular anomalies in 701 cases. Clin Transplant. (2001) 15(5):359–63. 10.1034/j.1399-0012.2001.150510.x 11678964

[25] Imam A Karatas C Mecit N KA Yildirimoglu T Kalayoglu M Anatomical variations of the hepatic artery: a closer view of rare unclassified variants. Folia Morphol (Praha). (2022) 81(2):359–64 10.5603/FM.a2021.0024 33749803

[26] De Blasi V Makkai-Popa S Arru L Pessaux P Azagra J. Rare anatomic variation of the hepatic arterial blood supply: case report and literature review. Surg Radiol Anat. (2019) 41(3):343–5. 10.1007/s00276-018-2163-5 30547210

[27] Darsan L Vishal V Cardoza F. Accessory right hepatic artery originating from proximal and distal right renal artery in two subjects. Indian J Urol. (2019) 35(4):305–6. 10.4103/iju.IJU_86_19 31619873 PMC6792413

[28] Al Zahrani Y AlMat’hami A Alobaidi H Wiseman D Mujoomdar A. Accessory right hepatic artery arising from splenic artery supplying hepatocellular carcinoma identified by computed tomography scan and conventional angiography: a rare anatomic variant. Ann Vasc Surg. (2017) 38(316):e1–e5. 10.1016/j.avsg.2016.05.096 27522974

[29] Caruso F Dondossola D Fornoni G Caccamo L Rossi G. Right hepatic artery from splenic artery: the four-leaf clover of hepatic surgery. Surg Radiol Anat. (2016) 38(7):867–71. 10.1007/s00276-016-1617-x 26769020

[30] Dandekar U Dandekar K Chavan S. Right hepatic artery: a cadaver investigation and its clinical significance. Anat Res Int. (2015):412595. 10.1155/2015/412595 26788371 PMC4695647

[31] Polguj M Gabryniak T Topol M. The right accessory hepatic artery; a case report and review of the literature. Surg Radiol Anat. (2010) 32(2):175–9. 10.1007/s00276-009-0536-5 19669076

[32] Wang K Hu S Jiang X Zhu M Jin B. Liver transplantation for patient with variant hepatic artery arising from right renal artery: a case report. Transplant Proc. (2007) 39(5):1716–7. 10.1016/j.transproceed.2006.11.008 17580230

[33] Abdullah S Mabrut J Garbit V De La Roche E Olagne E Rode A Anatomical variations of the hepatic artery: study of 932 cases in liver transplantation. Surg Radiol Anat. (2006) 28(5):468–73. 10.1007/s00276-006-0121-0 16642277

[34] Kishi Y Sugawara Y Kaneko J Akamatsu N Imamura H Asato H Hepatic arterial anatomy for right liver procurement from living donors. Liver Transpl. (2004) 10(1):129–33. 10.1002/lt.20010 14755789

[35] Braun M Collins M Wright P. An aberrant right hepatic artery from the right renal artery: anatomical vignette. Cardiovasc Intervent Radiol. (1991) 14(6):349–51. 10.1007/bf02577895 1756552

[36] Nakata K Higuchi R Ikenaga N Sakuma L Ban D Nagakawa Y Precision anatomy for safe approach to pancreatoduodenectomy for both open and minimally invasive procedure: a systematic review. J Hepatobiliary Pancreat Sci. (2022) 29(1):99–113. 10.1002/jhbp.901 33533158

[37] Montalti R Benedetti Cacciaguerra A Nicolini D Ahmed EA Coletta M De Pietri L Impact of aberrant left hepatic artery ligation on the outcome of liver transplantation. Liver Transpl. (2018) 24(2):204–13. 10.1002/lt.24992 29211941

[38] Ishigami K Zhang Y Rayhill S Katz D Stolpen A. Does variant hepatic artery anatomy in a liver transplant recipient increase the risk of hepatic artery complications after transplantation? AJR Am J Roentgenol. (2004) 183(6):1577–84. 10.2214/ajr.183.6.01831577 15547194

[39] Shukla PJ Barreto SG Kulkarni A Nagarajan G Fingerhut A. Vascular anomalies encountered during pancreatoduodenectomy: do they influence outcomes? Ann Surg Oncol. (2010) 17(1):186–93. 10.1245/s10434-009-0757-1 19838756

[40] Radtke A Sotiropoulos GC Molmenti EP Schroeder T Peitgen HO Frilling A Computer-assisted surgery planning for complex liver resections: when is it helpful? A single-center experience over an 8-year period. Ann Surg. (2010) 252(5):876–83. 10.1097/SLA.0b013e3181fdd012 21037445

[41] Crossingham JL Jenkinson J Woolridge N Gallinger S Tait GA Moulton C-AE. Interpreting three-dimensional structures from two-dimensional images: a web-based interactive 3d teaching model of surgical liver anatomy. HPB. (2009) 11(6):523–8. 10.1111/j.1477-2574.2009.00097.x 19816618 PMC2756641

